# Knowledge and Skills of Midwives Regarding Perinatal Mental Health and Their Needs for Further Education

**DOI:** 10.7759/cureus.76205

**Published:** 2024-12-22

**Authors:** Anastasia Karalia, Athina Diamanti, Christina I Nanou, Pinelopi Varela, Anna Deltsidou

**Affiliations:** 1 Department of Midwifery, University of West Attica, Athens, GRC

**Keywords:** education, mental illness, midwives, midwives' knowledge, perinatal mental health

## Abstract

Introduction

The literature highlights the crucial role of midwives in assessing and managing perinatal mental health and in providing information to women about related issues. However, research also indicates significant gaps in midwives' knowledge and skills needed to fulfill this role. Data suggest that while midwives are interested in providing mental health support, they lack the confidence, knowledge, and training to do so effectively. This study aimed to investigate the knowledge and skills of midwives regarding perinatal mental health, as well as the needs for further education in this area.

Methods

The study included 223 midwives from across Greece. Data were collected using a structured questionnaire with multiple sections. The first section included psychometric tools from the Professional Issues in Maternal Mental Health Scale (PIMMHS), assessing professional issues affecting midwifery practice. The second section used the Mental Illness Clinicians’ Attitudes Scale (MICA-4), and the third section used the Perinatal Mental Health Awareness (PMHA) scale. The final section evaluated midwives' learning needs for professional development. Data analysis was conducted using SPSS 22.0.

Results

The findings revealed significant gaps in midwives’ knowledge and confidence in managing perinatal mental health, particularly in areas such as bipolar disorder, schizophrenia, and anxiety disorders. The average knowledge score for perinatal mental health was 52.1%, indicating a moderate level of awareness, while the "Anxiety Worry and Depression" dimension of the PMHA scale had a mean score of 5.3 (range 4-6). Significant correlations were observed between midwives’ knowledge and their attitudes, with those possessing greater knowledge exhibiting less stigmatizing views toward mental illness (β = -0.004, p < 0.001). Midwives with higher scores in the "Emotion" dimension of the PIMMHS were also found to hold less negative attitudes toward mental illness (β = -0.01, p = 0.008). Additionally, midwives who frequently cared for women with mental illness had significantly higher knowledge scores (β = 0.18, p = 0.019). The majority of participants expressed a need for additional training, with 171 (76.7%) participants desiring more education on bipolar disorder and 164 (73.5%) on schizophrenia.

Conclusions

The results highlight significant knowledge gaps and management challenges among midwives in the area of perinatal mental health, primarily due to a lack of education. These findings underscore the need for training programs to improve midwives' knowledge and capabilities in perinatal mental health care.

## Introduction

The literature has highlighted significant risks for the presence of mental health issues during the perinatal period. Specifically, women with a prior history of bipolar disorder or those who have experienced previous postpartum psychosis are at an increased risk of psychosis relapse [1,2]. This risk is particularly elevated for those who abruptly discontinue medication [3]. Evidence suggests that a history of depression [4] or prenatal depression increases the risk of depression in the postpartum period [5]. Additionally, a prior history of anxiety disorders is a risk factor for postpartum mood disorders, such as depression or anxiety [6]. Studies have also emphasized the potential for recurrence or worsening of pre-existing conditions, such as binge eating disorder [7] and obsessive-compulsive disorder [8,9]. Furthermore, intimate partner violence rates often increase during pregnancy, with significant psychological consequences [10].

Women with a history of serious mental health issues, such as those diagnosed with schizophrenia, may delay seeking prenatal care due to fear of stigma, critical attitudes from healthcare staff, and concerns regarding child custody [11]. Studies also highlight the association between perinatal mental health problems and increased likelihood of difficult labor [12], as well as other complications such as preterm birth, low birth weight, stillbirth, and the need for postnatal specialized care for the infant [13,14]. Additionally, women receiving midwifery care have emphasized the need for more information on mental health and emotional well-being during prenatal care [15].

The existing literature underscores the significant role of midwives in the assessment and management of perinatal mental health, as well as the provision of information to women regarding these issues [15-17]. However, research has also revealed significant gaps in midwives' knowledge and skills to effectively fulfill this role [18-20]. The data suggest that while midwives are interested in providing mental health support, they lack the confidence, knowledge, and training necessary to do so [19,20].

A review of the Greek literature indicates that despite the importance of the topic, there is a lack of research on midwives’ knowledge and perceptions regarding perinatal mental health. In a study conducted in Cyprus, it was found that a large proportion of pregnant women and mothers experienced a decline in mental health due to the anxiety and changes brought about by pandemic-related protective measures in healthcare services [21]. Similarly, another study for Crete revealed that during the pandemic, the mental health needs of pregnant women and mothers of infants increased due to the greater challenges they faced [22]. In the same study, it was found that the provision of education to these women helped them effectively manage and maintain their mental health at a satisfactory level without the emergence of major issues [22]. Midwives managed the parental preparation program, highlighting their significant contribution to this field [22]. However, despite extensive investigation, no studies were identified that examined either the contribution of midwives or their attitudes and perceptions toward this issue. This gap in the Greek literature underscores the importance of conducting the present research, which aims to fill a significant void and provide recommendations for the training of midwives in this area.

## Materials and methods

Study design and sample selection

This is a cross-sectional quantitative study. The target population for this study was licensed midwives actively practicing in Greece, including those working in various healthcare settings such as public hospitals, private clinics, and community health centers. This population also encompassed midwives involved in different stages of maternity care, including prenatal, perinatal, and postnatal care services. Additionally, midwives practicing in both urban and rural regions were included to ensure representation of diverse healthcare contexts. This broad definition aimed to capture the varying levels of experience, training, and exposure to perinatal mental health challenges among midwives, providing a comprehensive understanding of their knowledge, attitudes, and educational needs in this field. The sample included midwives from all over Greece, and the total sample size was 223 participants.

The selection criteria for the sample were as follows: a) midwives who were willing to participate in the study and b) midwives who understood and spoke the Greek language.

The sampling method used was convenience and snowball sampling. Specifically, the sampling was conducted by posting an invitation to participate on social media (Facebook) in closed online groups of midwifery graduates and through the researcher’s personal communication network by sending emails to colleagues. Both the participation invitation and the email included a link redirecting participants to a specific platform with the online questionnaire.

Data collection tools

The data collection tool was a structured questionnaire consisting of five sections, each including different instruments (Appendix A). The inclusion of the instruments in the questionnaire followed communication with their creators and the receipt of the necessary permissions.

The first section included psychometric subscales from the Professional Issues in Maternal Mental Health Scale (PIMMHS), consisting of seven statements with responses on a five-point Likert scale. The PIMMHS is a psychometric tool designed to assess professional issues that affect midwifery practice. It is considered ideal for helping educational providers identify areas for curriculum development and for preventive evaluation of service delivery in maternity services, as well as for identifying training and service development opportunities [23].

The second section included the Mental Illness Clinicians' Attitudes Scale (MICA-4). This scale consists of 16 statements, with responses on a five-point Likert scale. The MICA-4 evaluates the attitudes of students and healthcare professionals toward individuals with mental illnesses. The scale has demonstrated good internal consistency (α = 0.72) and correlation among the items. The principal component analysis produced a five-factor structure, and the scale exhibited acceptable convergent validity [24].

The third section included the Perinatal Mental Health Awareness (stress, anxiety, and depression) [PMHA (SAD)] scale, consisting of three questions evaluating midwifery students' awareness of perinatal mental health. The scale allows for the assessment and comparison of different aspects of midwifery practice based on a holistic model of midwifery care [25].

The fourth section comprised a scale assessing midwives' learning needs for professional development, while also evaluating their knowledge and attitudes toward perinatal mental health. This scale was used in the study by Hauck et al. [17]. Finally, the fifth section covered the participants’ demographic and professional characteristics, consisting of seven questions in total.

Statistical analysis

Mean values and standard deviations (SDs) were used to describe the quantitative variables, while the median and interquartile range were used for the description of variables that did not follow a normal distribution and in cases where clearer descriptions of the variables were required. Absolute (N) and relative (%) frequencies were used for the description of qualitative variables. Normality checks of the distributions were conducted using the Kolmogorov-Smirnov criterion. Cronbach's α was used to assess the reliability of the factors that emerged from the data. The correlation coefficient of Spearman (rho) was used to assess the relationship between two quantitative variables. Linear regression analysis was employed to identify independent factors associated with the dimensions of the study scales, from which dependency coefficients (β) and their standard errors (SE) were derived. Multivariate linear regression analyses were conducted using logarithmic transformation of the dependent variable due to the absence of normality. Significance levels were two-tailed, with statistical significance set at 0.05. The statistical software SPSS Version 22.0 (IBM Corp., Armonk, NY) was used for the analysis.

Ethical approval

The research protocol received ethical approval from the Research Ethics and Deontology Committee of the University of West Attica (Approval No. 26056/14-03-2022). The committee confirmed compliance with ethical standards, legal regulations, and research integrity requirements, with the stipulation that any amendments to the protocol must be resubmitted for approval.

## Results

Demographics

The study sample consisted of 223 individuals. The demographic characteristics of the participants are presented in Table 1.

**Table 1:** Characteristics of the study population.

Characteristic	N	%
Age group		
20-25	24.0	10.8
26-30	46.0	20.6
31-35	37.0	16.6
36-40	36.0	16.1
41-45	39.0	17.5
46-50	23.0	10.3
51-55	11.0	4.9
55-60	7.0	3.1
60+	0.0	0.0
Years of experience as a midwife		
Less than one year	20.0	9.0
1-2	18.0	8.1
3-5	41.0	18.4
6-10	39.0	17.5
11-15	32.0	14.3
16-20	43.0	19.3
21-25	21.0	9.4
26-30	6.0	2.7
30+	3.0	1.3
How often do you care for a pregnant woman with mental illness?		
Never	59.0	26.5
Rarely	80.0	35.9
Sometimes	61.0	27.4
Often	16.0	7.2
Unsure	7.0	3.1
In which area of midwifery do you currently work?		
Prenatal services	31.0	13.9
Perinatal services	25.0	11.2
Postnatal services	23.0	10.3
Working in all these areas	144.0	64.6
Do you have work experience in mental health facilities?		
Yes	19.0	8.5
Have you participated in training in the last two years (seminar, workshop, online) in perinatal mental health?		
Yes	92.0	41.3
I feel well-trained to support women with perinatal mental disorders		
Yes	43.0	19.3
I feel I have sufficient access to information on mental health disorders during pregnancy and the perinatal period		
Yes	86.0	38.6

Intentions for additional training in mental disorders, knowledge areas, and skills

Intentions for additional training in mental disorders, knowledge areas, and skills are illustrated in Table 2.

**Table 2:** Intentions for additional training in mental disorders, knowledge areas, and skills.

Characteristic	N	%
For which of the following mental disorders would you like further training?		
Bipolar disorder	171.0	76.7
Schizophrenia	164.0	73.5
Personality disorders	154.0	69.1
Anxiety disorders	109.0	48.9
Tokophobia	96.0	43.0
Substance-related disorders	122.0	54.7
Depression	97.0	43.5
No further training needed	10.0	4.5
Other	1.0	0.4
In which of the following knowledge areas would you like further training?		
Signs and symptoms of mental illness	169.0	75.8
Roles of other health professionals	85.0	38.1
Understanding care options	152.0	68.2
Child safety	133.0	59.6
Impact of childbearing on mental health	138.0	61.9
No further training needed	12.0	5.4
Other	0.0	0.0
In which of the following skills would you like further training?		
Mental health assessment	157.0	70.4
Communication skills	106.0	47.5
Risk assessment for mental illness	123.0	55.2
Managing stress and aggression	139.0	62.3
Clinical management (medication, etc.)	135.0	60.5
Working with families and caregivers	99.0	44.4
Breastfeeding support	56.0	25.1
Hospital discharge planning	74.0	33.2
No further training needed	14.0	6.3
Other	1.0	0.4
Which form of training would you prefer?		
Series of workshops and seminars	170.0	76.2
Access to online packages	83.0	37.2
Seminar with guest speaker: half or full day (one cycle)	52.0	23.3
Workshop (active participation): half or full day (one cycle)	91.0	40.8
Other	1.0	0.4

Among the participants, 171 (76.7%) expressed a desire for further training on bipolar disorder, 164 (73.5%) on schizophrenia, and 154 (69.1%) on personality disorders. Additionally, 122 (54.7%) wanted more education on substance-related disorders and 109 (48.9%) on anxiety disorders. Furthermore, 97 (43.5%) sought additional training on depression and 96 (43.0%) on tokophobia, while 10 (4.5%) reported no need for further training.

In terms of knowledge areas, 169 (75.8%) sought training on recognizing the signs and symptoms of mental illness, 152 (68.2%) on understanding care options, and 138 (61.9%) on the impact of childbearing on mental health. Additionally, 133 (59.6%) expressed interest in training on child safety, while 85 (38.1%) wanted to learn about the roles of other health professionals. Only 12 (5.4%) felt no need for further training.

For skill development, 157 (70.4%) wanted to improve their mental health assessment skills, 139 (62.3%) sought training on managing stress and aggression, and 135 (60.5%) expressed interest in clinical management including medication. Moreover, 123 (55.2%) sought training in risk assessment for mental illness, while 99 (44.4%) wanted to enhance their ability to work with families and caregivers.

When asked about preferred training formats, 170 (76.2%) favored a series of workshops and seminars, 91 (40.8%) preferred a workshop involving active participation, and 83 (37.2%) selected access to online training packages. A smaller percentage (52, 23.3%) chose a one-time seminar with a guest speaker, and one (0.4%) indicated other preferences.

Psychometric retesting of the Professional Issues Scale for Midwives regarding maternal mental health

In Table 3, the psychometric retesting of the Professional Issues Scale for Midwives regarding maternal mental health is presented.

**Table 3:** Psychometric retesting of the Professional Issues Scale for Midwives regarding maternal mental health.

Statement	Strongly agree (N)	Strongly agree (%)	Agree (N)	Agree (%)	Neither agree nor disagree (N)	Neither agree nor disagree (%)	Disagree (N)	Disagree (%)	Strongly disagree (N)	Strongly disagree (%)
I know exactly whom to contact if a woman has mental health problems	55	24.7	75	33.6	72	32.3	14	6.3	7	3.1
Sometimes I feel reluctant to discuss emotional issues a woman might have because I feel uncomfortable discussing them with her	6	2.7	30	13.5	56	25.1	63	28.3	68	30.5
Sometimes I feel reluctant to discuss emotional issues a woman might have because I know I won’t have enough time to resolve them	14	6.3	32	14.3	80	35.9	54	24.2	43	19.3
Sometimes I feel reluctant to discuss emotional issues a woman might have because I don’t know what to do and whom to ask for advice	16	7.2	28	12.6	76	34.1	49	22.0	54	24.2
Sometimes I feel reluctant to discuss emotional issues a woman might have because I don’t feel adequately trained to address them	20	9.0	72	32.3	80	35.9	33	14.8	18	8.1
Training pays adequate attention to the cultural dimensions of pregnancy, childbirth, and postnatal care	40	17.9	62	27.8	78	35.0	29	13.0	14	6.3
It is easy for me to get help for women with mental health problems	31	13.9	48	21.5	104	46.6	32	14.3	8	3.6

Among the participants, 130 (58.3%) knew exactly whom to contact if a woman had mental health problems. Additionally, 131 (58.8%) disagreed or strongly disagreed that they sometimes felt reluctant to discuss emotional issues because they felt uncomfortable addressing them. Furthermore, 97 (43.5%) disagreed or strongly disagreed that they felt reluctant to discuss emotional issues due to a lack of time to resolve them.

Regarding specific barriers, 44 (19.8%) of participants agreed or strongly agreed that they felt reluctant to discuss emotional issues because they wouldn’t know what to do or whom to ask for advice, and 92 (41.3%) expressed reluctance due to feeling inadequately trained to address such issues. Moreover, 102 (45.7%) agreed or strongly agreed that training pays adequate attention to the cultural dimensions of pregnancy, childbirth, and postnatal care.

Lastly, 79 (35.4%) of participants agreed or strongly agreed that it was easy for them to get help for women with mental health problems.

Level of emotion and training for the PIMMHS psychometric retesting

Subsequently, the statements were appropriately coded and aggregated. This resulted in the scores of the dimensions of the PIMMHS psychometric retesting questionnaire. Higher scores in the dimensions are associated with more intense feelings and a higher level of training.

The score for the "Emotion" dimension of the Professional Issues Scale regarding PIMMHS ranged from 5 to 20 points. The mean score was 14.2 points (SD = 3.3 points), and the median score was 14 points (range = 12-16 points).

The score for the "Training" dimension of the PIMMHS scale ranged from 3 to 15 points. The mean score was 9.5 points (SD = 2.3 points), and the median score was 9 points (range = 8-11 points).

The Cronbach’s Alpha value for both dimensions was above the acceptable threshold (0.7).

Responses to the MICA-4 health professionals’ attitudes scale questions

In Table 4, the responses to the questions of the MICA-4 are presented.

**Table 4:** Responses to the MICA-4 health professionals’ attitudes scale questions. MICA-4, Mental Illness Clinicians’ Attitudes Scale

Statement	Strongly agree (N)	Strongly agree (%)	Agree (N)	Agree (%)	Somewhat agree (N)	Somewhat agree (%)	Somewhat disagree (N)	Somewhat disagree (%)	Disagree (N)	Disagree (%)	Strongly disagree (N)	Strongly disagree (%)
I learn about mental health only when necessary and would not take the time to study additional material on the subject	3	1.3	23	10.3	34	15.2	40	17.9	53	23.8	70	31.4
People with severe mental illness can never fully recover to have a good quality of life	1	0.4	6	2.7	26	11.7	37	16.6	56	25.1	97	43.5
Work in mental health is just as respected as in other health and social care sectors	185	83.0	23	10.3	8	3.6	4	1.8	1	0.4	2	0.9
If I had a mental illness, I wouldn’t admit it to those close to me because I would be afraid they would treat me differently	2	0.9	12	5.4	29	13.0	26	11.7	50	22.4	104	46.6
People with severe mental illness are more likely to be dangerous than those without mental illness	35	15.7	43	19.3	60	26.9	39	17.5	31	13.9	15	6.7
Health and social care professionals know more about the lives of those they care for than their relatives or friends	54	24.2	60	26.9	68	30.5	23	10.3	13	5.8	5	2.2
If I had a mental illness, I would never admit it to my colleagues for fear they would treat me differently	9	4.0	33	14.8	54	24.2	28	12.6	35	15.7	64	28.7
Being a health/social care professional in mental health is not like being a traditional healthcare professional	18	8.1	34	15.2	33	14.8	37	16.6	41	18.4	60	26.9
If a superior colleague instructed me to treat people with mental illness disrespectfully, I would not follow the instructions	153	68.6	32	14.3	11	4.9	9	4.0	10	4.5	8	3.6
I feel just as comfortable talking to someone with a mental illness as I do with someone with a physical illness	71	31.8	61	27.4	50	22.4	29	13.0	9	4.0	3	1.3
It is important that when a health/social care professional supports a person with a mental illness, they ensure that their physical health is also evaluated	149	66.8	53	23.8	14	6.3	6	2.7	0	0.0	1	0.4
People in our society do not need protection from those with severe mental illness	11	4.9	24	10.8	69	30.9	49	22.0	24	10.8	46	20.6
If someone with a mental illness reported physical symptoms (like chest pain), I would attribute them to their mental illness	3	1.3	9	4.0	30	13.5	38	17.0	55	24.7	88	39.5
General practitioners should not be expected to complete thorough assessments for people with psychiatric symptoms because they can be referred to a psychiatrist	47	21.1	40	17.9	65	29.1	31	13.9	17	7.6	23	10.3
I would use terms like "crazy," "lunatic," or "mad" to describe to colleagues people with mental illness I have met at work	3	1.3	8	3.6	20	9.0	17	7.6	21	9.4	154	69.1
If a colleague told me they had a mental illness, I would continue to work with them	116	52.0	51	22.9	35	15.7	13	5.8	6	2.7	2	0.9

Among the participants, 70 (31.4%) strongly disagreed with the statement that they learn about mental health only when necessary and would not take the time to study additional material, while 97 (43.5%) strongly disagreed that people with severe mental illness can never fully recover to have a good quality of life. Additionally, 185 (83.0%) strongly agreed that work in mental health is just as respected as in other health and social care sectors, but 104 (46.6%) strongly disagreed with admitting to close ones that they had a mental illness for fear of being treated differently.

Regarding specific beliefs and attitudes, 60 (26.9%) somewhat agreed that people with severe mental illness are more likely to be dangerous compared to those without mental illness, 68 (30.5%) somewhat agreed that health and social care professionals know more about the lives of those they care for than their relatives or friends, 64 (28.7%) strongly disagreed with the statement that they would never admit to colleagues that they had a mental illness for fear of being treated differently, and 60 (26.9%) strongly disagreed with the notion that being a health/social care professional in mental health is not like being a traditional healthcare professional.

In terms of professional conduct and values, 153 (68.6%) strongly agreed that if a superior instructed them to treat people with mental illness disrespectfully, they would not follow such instructions, 71 (31.8%) strongly agreed that they felt just as comfortable talking to someone with a mental illness as with someone with a physical illness, and 149 (66.8%) strongly agreed that when supporting a person with a mental illness, it is important to ensure their physical health is also evaluated.

When addressing societal attitudes, 46 (20.6%) strongly disagreed that people in society do not need protection from those with severe mental illness and 88 (39.5%) strongly disagreed with attributing physical symptoms (such as chest pain) to mental illness.

Regarding general practitioner responsibilities, 47 (21.1%) strongly agreed that general practitioners should not be expected to complete thorough assessments for psychiatric symptoms since they can refer patients to a psychiatrist and 154 (69.1%) strongly disagreed with using terms such as "crazy," "lunatic," or "mad" to describe individuals with mental illness to colleagues.

Overall score for the Health Professionals’ Attitudes Scale for Mental Illness (MICA-4)

Subsequently, the statements were appropriately coded and aggregated. This resulted in the dimensions and overall score of the MICA-4. A higher score indicates more negative, stigmatizing attitudes toward mental illness.

The overall score of the Health Professionals' Attitudes Scale for Mental Illness (MICA-4) ranged from 20 to 63 points. The mean score was 41.4 points (SD = 8.6 points), and the median score was 41 points (range = 35-48 points). The score for the "Views on health/social care and mental illness" dimension of the MICA-4 ranged from 4 to 17 points. The mean score was 9.3 points (SD = 2.7 points), and the median score was 9 points (range = 7-11 points).

The score for the "Knowledge of mental illness" dimension of the MICA-4 ranged from 5 to 23 points. The mean score was 12.9 points (SD = 2.9 points), and the median score was 13 points (range = 11-15 points). The score for the "Disclosure" dimension of the MICA-4 ranged from 2 to 12 points. The mean score was 5 points (SD = 2.5 points), and the median score was 5 points (range = 2-7 points).

The score for the "Distinction between mental and physical health" dimension of the MICA-4 ranges from 4 to 22 points. The mean score was 10.9 points (SD = 3.4 points), and the median score was 10 points (range = 9-13 points). The score for the "Patient care for people with mental illness" dimension of the MICA-4 ranged from 2 to 11 points. The mean score was 3.2 points (SD = 1.8 points), and the median score was 2 points (range = 2-4 points).

The Cronbach's alpha values for all dimensions were above the acceptable threshold (0.7).

Spearman correlation coefficients (rho) between the participants' scores on the PIMMHS and the MICA-4

In Table 5, the Spearman correlation coefficients (rho) between the participants' scores on the PIMMHS and the MICA-4 are presented.

**Table 5:** Correlation between the Maternal Mental Health Professional Issues Scale and the Health Professionals’ Attitudes Scale for Mental Illness (MICA-4). MICA-4, Mental Illness Clinicians’ Attitudes Scale

Dimension	Emotion (rho)	Emotion (P)	Training (rho)	Training (P)
Views on health/social care and mental illness	-0.09	0.184	-0.18	0.006
Knowledge of mental illness	-0.24	<0.001	-0.1	0.132
Disclosure	-0.1	0.119	-0.04	0.579
Distinction between mental and physical health	-0.13	0.059	-0.02	0.758
Patient care for people with mental illness	0.02	0.74	-0.03	0.633
Overall Health Professionals’ Attitudes Scale for Mental Illness (MICA-4)	-0.19	0.004	-0.11	0.091

The "Emotion" dimension of the PIMMHS was found to be negatively correlated with the "Knowledge of mental illness" dimension and the overall MICA-4. Specifically, the more intense the feelings related to maternal mental health issues, the less negative were the participants' stigmatizing attitudes toward mental illness and psychiatry.

The "Training" dimension of the PIMMHS was found to be negatively correlated with the "Views on health/social care and mental illness" dimension of the MICA-4. More specifically, the higher the participants' level of training on maternal mental health, the less negative their stigmatizing views toward health, social care, and mental illness.

Responses to the PMHA

In Table 6, the responses to the PMHA are presented.

**Table 6:** Level of knowledge, recognition, and management of conditions related to anxiety, worry and depression, learning difficulties, and medical and obstetric conditions such as HIV and pubic symphysis separation

Condition	Not at all knowledgeable (N)	Not at all knowledgeable (%)	Not very knowledgeable (N)	Not very knowledgeable (%)	Knowledgeable (N)	Knowledgeable (%)	Very knowledgeable (N)	Very knowledgeable (%)
Anxiety, worry, and depression	8	3.6	41	18.4	130	58.3	44	19.7
Learning difficulties	44	19.7	95	42.6	68	30.5	16	7.2
Medical, obstetric conditions (e.g., preeclampsia, HIV, pubic symphysis separation)	2	0.9	12	5.4	95	42.6	114	51.1
Anxiety, worry, and depression (recognition)	8	3.6	56	25.1	118	52.9	41	18.4
Learning difficulties (recognition)	46	20.6	100	44.8	65	29.1	12	5.4
Medical, obstetric conditions (recognition)	2	0.9	20	9.0	105	47.1	96	43.0
Anxiety, worry, and depression (management)	17	7.6	100	44.8	85	38.1	21	9.4
Learning difficulties (management)	77	34.5	94	42.2	47	21.1	5	2.2
Medical, obstetric conditions (management)	2	0.9	25	11.2	113	50.7	83	37.2

Among the participants, 174 (78.0%) considered themselves knowledgeable or very knowledgeable about conditions related to anxiety, worry, and depression, whereas 139 (62.3%) did not consider themselves knowledgeable about learning difficulties. Additionally, 159 (71.3%) reported feeling sure or very sure about recognizing conditions related to anxiety, worry, and depression. An even higher percentage, 201 (90.1%), expressed confidence in recognizing medical or obstetric conditions such as preeclampsia, HIV, and pubic symphysis separation.

Regarding anxiety, worry, and depression, 130 (58.3%) were knowledgeable and 44 (19.7%) were very knowledgeable. For learning difficulties, 44 (19.7%) were not at all knowledgeable and 95 (42.6%) were not very knowledgeable. Regarding medical or obstetric conditions, 95 (42.6%) felt knowledgeable and 114 (51.1%) felt very knowledgeable.

In addition, 118 (52.9%) were sure that they could recognize conditions related to anxiety, worry, and depression, while 41 (18.4%) were very sure. Confidence in recognizing learning difficulties was notably lower, with 100 (44.8%) not very sure and 46 (20.6%) not at all sure. In contrast, 105 (47.1%) felt sure about recognizing medical or obstetric conditions and 96 (43.0%) were very sure.

Only 106 (47.5%) felt sure or very sure about managing anxiety, worry, and depression, with 17 (7.6%) reporting not at all sure. For learning difficulties, 171 (76.7%) felt not at all sure or not very sure about management, highlighting a considerable gap in confidence. On the other hand, 196 (87.9%) were sure or very sure about managing medical or obstetric conditions, with 113 (50.7%) feeling sure and 83 (37.2%) very sure.

Overall score for PMHA

The statements were appropriately coded and summed, leading to the creation of the dimensions and the overall score for the PMHA. A higher score indicates better knowledge, management, and organization of situations related to PMHA.

The score for the "Anxiety, worry, and depression" dimension of the PMHA ranged from 0 to 9 points. The mean score was 5.3 points (SD = 2 points), and the median score was 5 points (range = 4-6 points).

The score for the "Learning difficulties" dimension of the PMHA ranged from 0 to 9 points. The mean score was 3.4 points (SD = 2.3 points), and the median score was 3 points (range = 2-5 points).

The score for the "Medical, obstetric conditions (e.g., preeclampsia, HIV, pubic symphysis separation)" dimension of the PMHA ranged from 0 to 9 points. The mean score was 7 points (SD = 1.9 points), and the median score was 7 points (range = 6-9 points).

The overall score for the PMHA ranged from 6 to 27 points. The mean score was 15.7 points (SD = 4.2 points), and the median score was 15 points (range = 13-18 points). The Cronbach's Alpha value was above the acceptable threshold (0.7).

Spearman correlation coefficients (rho) between the participants' scores on the PMHA and the scores from the PIMMHS and the MICA-4

Ιn Table 7, the Spearman correlation coefficients (rho) between the participants' scores on the PMHA and the scores from the PIMMHS and the MICA-4 are presented.

**Table 7:** Spearman correlation coefficients between the PMHA scale, the Maternal Mental Health Professional Issues Scale (PIMMHS), and the Health Professionals’ Attitudes Scale for Mental Illness (MICA-4). PMHA, Perinatal Mental Health Awareness Scale; PIMMHS, Professional Issues in Maternal Mental Health Scale; MICA-4, Mental Illness Clinicians’ Attitudes Scale

Dimension	Anxiety, worry, and depression (rho)	Anxiety, worry, and depression (P)	Learning difficulties (rho)	Learning difficulties (P)	Medical, obstetric conditions (rho)	Medical, obstetric conditions (P)	Overall PMHA scale (rho)	Overall PMHA scale (P)
Maternal Mental Health Professional Issues Scale (PIMMHS): Emotion	0.33	<0.001	0.25	<0.001	0.16	0.02	0.35	<0.001
Maternal Mental Health Professional Issues Scale (PIMMHS): Training	0.29	<0.001	0.31	<0.001	0.21	0.002	0.36	<0.001
Health Professionals’ Attitudes Scale for Mental Illness (MICA-4): Views on health/social care and mental illness	-0.18	0.008	-0.08	0.251	-0.11	0.116	-0.17	0.01
Health Professionals’ Attitudes Scale for Mental Illness (MICA-4): Knowledge of mental illness	-0.16	0.015	-0.01	0.838	-0.02	0.711	-0.1	0.14
Health Professionals’ Attitudes Scale for Mental Illness (MICA-4): Disclosure	-0.07	0.333	0.09	0.16	-0.3	<0.001	-0.08	0.223
Health Professionals’ Attitudes Scale for Mental Illness (MICA-4): Distinction between mental and physical health	-0.02	0.751	0.09	0.166	-0.21	0.002	-0.05	0.465
Health Professionals’ Attitudes Scale for Mental Illness (MICA-4): Patient care for people with mental illness	-0.01	0.851	0.12	0.083	-0.16	0.02	0.001	0.976
Health Professionals’ Attitudes Scale for Mental Illness (MICA-4): Overall scale	-0.14	0.035	0.07	0.332	-0.24	<0.001	-0.12	0.076

The scores for the dimensions and the overall PMHA were found to be positively correlated with the dimensions of the PIMMHS. Specifically, stronger emotions and a higher level of training among the participants in maternal mental health issues were associated with better knowledge regarding the management and organization of situations related to perinatal mental health. Furthermore, the score for the "Anxiety, worry, and depression" dimension of the PMHA scale was negatively correlated with the scores for the "Views on health/social care and mental illness" and "Knowledge of mental illness" dimensions, as well as with the overall score of the MICA-4. Specifically, the better the participants' knowledge regarding the management and organization of anxiety, worry, and depression, the less negative stigmatizing attitudes they exhibited toward mental illness and psychiatry.

Additionally, the score for the "Medical, obstetric conditions (e.g., preeclampsia, HIV, pubic symphysis separation)" dimension of the PMHA scale was negatively correlated with the "Disclosure" and "Distinction between mental and physical health" dimensions, as well as with the overall MICA-4.

Finally, the overall PMHA was negatively correlated with the score for the "Views on health/social care and mental illness" dimension of the MICA-4.

Responses regarding the learning needs of midwives for professional development along with the assessment of their knowledge of perinatal mental health

In Table 8, the responses regarding the learning needs of midwives for professional development are presented, along with the assessment of their knowledge of perinatal mental health.

**Table 8:** Midwives' learning needs for professional development with concurrent assessment of their knowledge of perinatal mental health. Note: + denotes the correct answers EPDS, Edinburgh Postnatal Depression Scale

Question	Strongly agree (N)	Strongly agree (%)	Agree (N)	Agree (%)	Neutral (N)	Neutral (%)	Disagree (N)	Disagree (%)	Strongly disagree (N)
Hormones secreted during pregnancy protect against mental illness+	0	0.0	13	5.8	56	25.1	70	31.4	84
Pregnant women should not take psychiatric medication+	5	2.2	20	9.0	71	31.8	70	31.4	57
Women should not breastfeed if they are taking medication for mental illness+	5	2.2	31	13.9	74	33.2	69	30.9	44
The cut-off score on the EPDS that suggests further evaluation is 9+	39	17.5	47	21.1	91	40.8	27	12.1	19
Women with mental illness may have attachment problems with their newborn+	57	25.6	93	41.7	60	26.9	12	5.4	1
A mental illness during pregnancy can affect the newborn’s birth weight+	34	15.2	77	34.5	86	38.6	19	8.5	7
Family history of mental illness is not a risk factor for developing mental illness during pregnancy+	6	2.7	9	4.0	40	17.9	78	35.0	90
The EPDS is a useful diagnostic tool for depression and anxiety+	99	44.4	66	29.6	48	21.5	9	4.0	1
Women with postpartum depression are sad and cry all the time+	7	3.1	33	14.8	62	27.8	62	27.8	59
A previous history of mental illness is a risk factor for developing mental illness during pregnancy+	109	48.9	86	38.6	23	10.3	4	1.8	1
Postpartum depression will go away on its own but sometimes requires treatment+	10	4.5	53	23.8	25	11.2	40	17.9	95
Younger mothers are at higher risk of developing postpartum depression+	14	6.3	45	20.2	105	47.1	26	11.7	33
A history of abuse and trauma may increase the risk of developing mental illness in women+	133	59.6	66	29.6	20	9.0	3	1.3	1

Overall, 154 (69.1%) of participants disagreed or strongly disagreed with the statement that hormones secreted during pregnancy protect against mental illness. Similarly, 127 (57.0%) disagreed or strongly disagreed with the notion that pregnant women should not take psychiatric medication. Additionally, 69 (30.9%) disagreed that women should not breastfeed while taking medication for mental illness, reflecting an awareness of the compatibility of psychiatric treatment with breastfeeding.

Moreover, 150 (67.3%) of participants agreed or strongly agreed that women with mental illness may have attachment problems with their newborns, and 111 (49.7%) agreed or strongly agreed that mental illness during pregnancy could affect the newborn's birth weight.

Also, 195 (87.5%) and 199 (89.2%) of participants agreed or strongly agreed that both a previous history of mental illness and a history of abuse or trauma, respectively, increase the risk of developing mental illness during pregnancy. Furthermore, 135 (60.5%) disagreed or strongly disagreed with the notion that postpartum depression will go away on its own, recognizing the need for treatment in such cases.

Regarding specific beliefs, 91 (40.8%) of participants were neutral about the cut-off score of 9 on the Edinburgh Postnatal Depression Scale (EPDS) suggesting further evaluation, 99 (44.4%) strongly agreed that the EPDS is a useful diagnostic tool for depression and anxiety, and 133 (59.6%) strongly agreed that a history of abuse and trauma increases the risk of developing mental illness. Only seven (3.1%) strongly agreed that women with postpartum depression are sad and cry all the time, indicating an understanding of the nuanced presentation of postpartum depression. Lastly, 59 (26.5%) disagreed or strongly disagreed that younger mothers are at higher risk of developing postpartum depression, while 105 (47.1%) were neutral on this statement, highlighting some uncertainty in this area.

Percentages of correct and incorrect answers regarding their learning needs for professional development, along with an assessment of their knowledge of perinatal mental health

In Table 9, the percentages of correct and incorrect answers by midwives regarding their learning needs for professional development, along with an assessment of their knowledge of perinatal mental health, are presented.

**Table 9:** Percentage of correct/incorrect answers by midwives regarding their learning needs for professional development with concurrent assessment of their knowledge of perinatal mental health. EPDS, Edinburgh Postnatal Depression Scale

Question	Wrong (N)	Wrong (%)	Correct (N)	Correct (%)	Ambiguous (N)	Ambiguous (%)
Hormones secreted during pregnancy protect against mental illness	13	5.8	154	69.1	56	25.1
Pregnant women should not take psychiatric medication	25	11.2	127	57.0	71	31.8
Women should not breastfeed if they are taking medication for mental illness	36	16.1	113	50.7	74	33.2
The cut-off score on the EPDS that suggests further evaluation is 9	86	38.6	46	20.6	91	40.8
Women with mental illness may have attachment problems with their newborn	13	5.8	150	67.3	60	26.9
A mental illness during pregnancy can affect the newborn’s birth weight	111	49.8	26	11.7	86	38.6
Family history of mental illness is not a risk factor for developing mental illness during pregnancy	15	6.7	168	75.3	40	17.9
The EPDS is a useful diagnostic tool for depression and anxiety	165	74.0	10	4.5	48	21.5
Women with postpartum depression are sad and cry all the time	40	17.9	121	54.3	62	27.8
A previous history of mental illness is a risk factor for developing mental illness during pregnancy	5	2.2	195	87.4	23	10.3
Postpartum depression will go away on its own but sometimes requires treatment	63	28.3	135	60.5	25	11.2
Younger mothers are at higher risk of developing postpartum depression	59	26.5	59	26.5	105	47.1
A history of abuse and trauma may increase the risk of developing mental illness in women	4	1.8	199	89.2	20	9.0

The analysis of participants' responses revealed that the statements “A previous history of mental illness is a risk factor for developing mental illness during pregnancy” and “A history of abuse and trauma may increase the risk of developing mental illness in women” had the highest percentages of correct answers, with success rates of 195 (87.4%) and 199 (89.2%), respectively. These findings suggest a strong understanding of key risk factors for mental illness during pregnancy.

Conversely, the statement “The Edinburgh Postnatal Depression Scale (EPDS) is a useful diagnostic tool for depression and anxiety” had the highest percentage of incorrect answers, with 165 (74.0%) responding incorrectly, indicating a notable gap in knowledge regarding the use of this tool. Ambiguity was most prevalent for the statements “Younger mothers are at higher risk of developing postpartum depression” and “The cut-off score on the EPDS that suggests further evaluation is 9,” with 105 (47.1%) and 91 (40.8%) of responses classified as ambiguous, respectively. This highlights areas where participants may lack clarity or certainty.

Additional findings show that 154 (69.1%) correctly identified that hormones secreted during pregnancy do not protect against mental illness, while 127 (57.0%) correctly disagreed with the misconception that pregnant women should not take psychiatric medication. Furthermore, 113 (50.7%) correctly responded that women can breastfeed while taking medication for mental illness, though 74 (33.2%) were ambiguous in their responses.

Misunderstandings were also observed regarding the impact of mental illness on the newborn's birth weight, with only 26 (11.7%) answering correctly and 111 (49.8%) responding incorrectly. Similarly, while 121 (54.3%) correctly disagreed with the oversimplified portrayal of postpartum depression as only involving sadness and crying, 40 (17.9%) responded incorrectly, indicating a partial understanding of the condition.

Percentage knowledge score of midwives regarding perinatal mental health

Subsequently, the 13 questions regarding the participants’ knowledge of perinatal mental health were coded appropriately (correct answers were assigned a value of 1 and incorrect or ungraded answers were assigned a value of 0). These were summed and transformed into a percentage scale to create the "Knowledge Score," which ranged from 0 to 100 points. A higher score in the “Knowledge” factor indicated better knowledge of perinatal mental health among the participants.

The percentage knowledge score of midwives regarding perinatal mental health ranged from 7.7 to 84.6 points. The mean score was 52.1 points (SD = 16.3 points), and the median score was 53.8 points (range = 38.5-61.5 points).

Midwives' learning needs for professional development

In Table 10, the responses regarding midwives' learning needs for professional development are presented, along with an evaluation of their attitudes toward perinatal mental health.

**Table 10:** Percentage of correct/incorrect answers by midwives regarding their learning needs for professional development with concurrent assessment of their attitudes on perinatal mental health.

Question	Strongly agree (N)	Strongly agree (%)	Agree (N)	Agree (%)	Neutral (N)	Neutral (%)	Disagree (N)	Disagree (%)	Strongly disagree (%)
It is easy to identify mental health problems in pregnant women	18	8.1	73	32.7	113	50.7	18	8.1	0.4
Pregnant women with mental health issues during pregnancy should always be referred to a specialist	156	70.0	54	24.2	6	2.7	7	3.1	0.0
Pregnant women suffering from mental illness are likely to be difficult to manage	57	25.6	87	39.0	65	29.1	12	5.4	0.9
Women with mental illness are unlikely to recover	3	1.3	15	6.7	31	13.9	78	35.0	43.0
It is not the midwife's responsibility to assess the mental health of a woman under their care	4	1.8	1	0.4	28	12.6	61	27.4	57.8
Women with severe mental illness should not be encouraged to have children	12	5.4	21	9.4	82	36.8	51	22.9	25.6
Women with severe mental illness should not be allowed to hold their newborns	7	3.1	31	13.9	76	34.1	67	30.0	18.8

Among the participants, 91 (40.8%) believed that it was easy to identify mental health problems in pregnant women, with 18 (8.1%) strongly agreeing and 73 (32.7%) agreeing with the statement. However, a notable 113 (50.7%) were neutral on this topic, suggesting uncertainty in identifying such issues. Nearly all participants (210, 94.2%) agreed or strongly agreed that pregnant women with mental health issues during pregnancy should always be referred to a specialist, reflecting strong consensus on the importance of specialist involvement.

Challenges of Managing Mental Illness

Overall, 144 (64.6%) agreed or strongly agreed that pregnant women suffering from mental illness are likely to be difficult to manage, while 65 (29.1%) remained neutral on the matter.

Recovery Prospects

Of the participants, 95 (43.0%) strongly disagreed with the statement that women with mental illness are unlikely to recover, and an additional 78 (35.0%) disagreed, indicating a generally optimistic view on recovery prospects.

Similarly, 108 (48.8%) disagreed or strongly disagreed with the statement that women with severe mental illness should not be allowed to hold their newborns, while 76 (34.1%) were neutral, highlighting some variability in attitudes.

Reproductive Rights and Responsibilities

Regarding the statement that women with severe mental illness should not be encouraged to have children, 82 (36.8%) were neutral, suggesting ambivalence, while 108 (48.5%) disagreed or strongly disagreed.

Additionally, 128 (57.8%) strongly disagreed with the notion that it is not the midwife's responsibility to assess the mental health of a woman under their care, showing strong agreement on the midwife's role in mental health care.

Score for midwives' attitudes regarding perinatal mental health

Subsequently, the seven questions regarding the participants' attitudes toward perinatal mental health were coded and summed, resulting in the attitude score for perinatal mental health. A higher score on the "Attitudes" factor indicates a more positive attitude toward pregnant women with mental health issues.

The "Attitudes" dimension score from the knowledge and attitudes scale of midwives regarding perinatal mental health ranged from 17 to 35 points. The mean score was 27.4 points (SD = 3.1 points), and the median score was 27 points (range = 25-30 points).

A higher score in the "Attitudes" factor indicates a more positive attitude toward pregnant women with mental health issues.

Responses regarding how midwives perceive different groups of pregnant women

In Table 11, the responses regarding how midwives perceive different groups of pregnant women are presented.

**Table 11:** Midwives' perceptions of different groups of pregnant women.

Question	Not at all (N)	Not at all (%)	A little (N)	A little (%)	Quite (N)	Quite (%)	Very (N)	Very (%)	Extremely (N)	Extremely (%)
How capable and skilled are pregnant women who also manage a household?	4	1.8	10	4.5	71	31.8	61	27.4	77	34.5
How warm and friendly are pregnant women who also manage a household?	2	0.9	14	6.3	82	36.8	56	25.1	69	30.9
How capable and skilled are pregnant women with a physical disability?	21	9.4	71	31.8	76	34.1	43	19.3	12	5.4
How warm and friendly are pregnant women with a physical disability?	5	2.2	29	13.0	86	38.6	62	27.8	41	18.4
How capable and skilled are pregnant women who are dependent on drugs?	117	52.5	84	37.7	11	4.9	8	3.6	3	1.3
How warm and friendly are pregnant women who are dependent on drugs?	105	47.1	84	37.7	19	8.5	12	5.4	3	1.3
How capable and skilled are pregnant women who run their own business?	2	0.9	18	8.1	48	21.5	72	32.3	83	37.2
How warm and friendly are pregnant women who run their own business?	2	0.9	25	11.2	59	26.5	61	27.4	76	34.1
How capable and skilled are pregnant women with bipolar disorder?	60	26.9	93	41.7	55	24.7	13	5.8	2	0.9
How warm and friendly are pregnant women with bipolar disorder?	57	25.6	85	38.1	67	30.0	13	5.8	1	0.4
How capable and skilled are pregnant women with anxiety?	16	7.2	106	47.5	63	28.3	34	15.2	4	1.8
How warm and friendly are pregnant women with anxiety?	21	9.4	108	48.4	64	28.7	26	11.7	4	1.8
How capable and skilled are pregnant women with housing issues?	50	22.4	64	28.7	77	34.5	25	11.2	7	3.1
How warm and friendly are pregnant women with housing issues?	30	13.5	60	26.9	83	37.2	39	17.5	11	4.9
How capable and skilled are pregnant women with depression?	83	37.2	85	38.1	37	16.6	14	6.3	4	1.8
How warm and friendly are pregnant women with depression?	84	37.7	87	39.0	33	14.8	17	7.6	2	0.9
How capable and skilled are pregnant women with schizophrenia?	138	61.9	55	24.7	18	8.1	7	3.1	5	2.2
How warm and friendly are pregnant women with schizophrenia?	136	61.0	59	26.5	17	7.6	6	2.7	5	2.2

Among participants, 77 (34.5%) believed that most midwives consider pregnant women who manage a household as extremely capable and skilled, while 82 (36.8%) thought they are viewed as quite warm and friendly. Conversely, 71 (31.8%) of participants thought that most midwives see pregnant women with physical disabilities as only a little capable and skilled, while 86 (38.6%) believed they are considered quite warm and friendly.

Pregnant women who are drug-dependent were perceived more negatively, with 117 (52.5%) of participants believing that most midwives view them as not at all capable or skilled and 105 (47.1%) believing that they are not seen as warm or friendly. On the other hand, 83 (37.2%) of the sample thought that most midwives consider pregnant women who run their own business as extremely capable and skilled, and 76 (34.1%) believed that these women are considered extremely warm and friendly.

Regarding mental health conditions, 93 (41.7%) of participants believed that most midwives view pregnant women with bipolar disorder as only a little capable and skilled, with 85 (38.1%) holding similar views about their warmth and friendliness. Likewise, 106 (47.5%) thought that most midwives consider pregnant women with anxiety as only a little capable and skilled, while 108 (48.4%) felt that they are also considered only a little warm and friendly.

Pregnant women facing social challenges also faced mixed perceptions. About 77 (34.5%) of participants believed that most midwives view pregnant women with housing issues as quite capable, skilled, warm, and friendly. However, around 87 (39.0%) thought that most midwives perceive pregnant women with depression as only a little capable, skilled, warm, and friendly. Also, 138 (61.0%) of participants believed that most midwives view pregnant women with schizophrenia as not at all capable, skilled, warm, or friendly, highlighting the most negative perceptions among all categories.

Midwives' perceptions of how capable, warm, friendly, and skilled pregnant women are, according to other midwives

Subsequently, the above questions were appropriately coded and summed, resulting in a score reflecting how midwives perceive different groups of pregnant women. A higher score indicates a stronger belief in how capable, warm, friendly, and skilled midwives perceive pregnant women to be.

The score reflecting midwives' perceptions of different groups of pregnant women ranged from 18 to 82 points. The mean score was 46.8 points (SD = 9 points), and the median score was 46 points (range = 41-51 points). The Cronbach's alpha value was above the acceptable threshold (0.7).

Correlation of the Perinatal Mental Health Awareness Scale, the Professional Issues in Maternal Mental Health Scale, and the Mental Illness Clinicians' Attitudes Scale with knowledge, attitudes, and perceptions of other midwives toward different groups of pregnant women

Subsequently, the Spearman correlation coefficients (rho) between participants' scores on the knowledge and attitudes scale for perinatal mental health and their perceptions of different groups of pregnant women were calculated. The correlations also included participants' scores on the PMHA, the PIMMHS, and the MICA-4. The attitude scores of participants toward perinatal mental health were positively correlated with the "Anxiety, worry, and depression" and "Medical, obstetric conditions (e.g., preeclampsia, HIV, symphysis pubis dysfunction)" dimensions, as well as with the overall PMHA score. In contrast, these scores were negatively correlated with the dimensions "Opinions on health, social care, and mental illness," "Disclosure," "Distinction between mental and physical health," and "Care for patients with mental illness," as well as with the overall MICA-4 score (Table 12).

**Table 12:** Correlation of the PMHA Scale, PIMMHS, and MICA-4 Scale with knowledge, attitudes, and perceptions of other midwives toward different groups of pregnant women. PMHA, Perinatal Mental Health Awareness; PIMMHS, Professional Issues in Maternal Mental Health Scale; MICA-4, Mental Illness Clinicians' Attitudes Scale

Scale	Knowledge score (%)	Attitude score	Perceptions of other midwives on different groups of pregnant women
PMHA	rho = 0.02, P = 0.735	rho = 0.21, P = 0.002	rho = -0.08, P = 0.259
Anxiety, worry, and depression	rho = -0.20, P = 0.003	rho = 0.06, P = 0.370	rho = 0.001, P = 0.989
Learning disabilities	rho = 0.08, P = 0.229	rho = 0.17, P = 0.010	rho = 0.001, P = 0.984
Medical, obstetric conditions (e.g., preeclampsia, HIV, symphysis pubis dysfunction)	rho = -0.08, P = 0.209	rho = 0.19, P = 0.004	rho = -0.06, P = 0.352
Overall PMHA score	rho = -0.08, P = 0.222	rho = 0.07, P = 0.279	rho = 0.07, P = 0.270
PIMMHS	rho = -0.11, P = 0.131	rho = 0.07, P = 0.287	rho = -0.03, P = 0.670
Emotion	rho = -0.16, P = 0.015	rho = -0.28, P < 0.001	rho = 0.11, P = 0.131
Education	rho = -0.08, P = 0.221	rho = -0.13, P = 0.057	rho = 0.02, P = 0.720
MICA-4	rho = -0.28, P < 0.001	rho = -0.28, P < 0.001	rho = 0.10, P = 0.118
Opinions on health, social care, and mental illness	rho = -0.29, P < 0.001	rho = -0.27, P < 0.001	rho = 0.08, P = 0.254
Knowledge of mental illness	rho = -0.27, P < 0.001	rho = -0.27, P < 0.001	rho = 0.02, P = 0.757
Disclosure	rho = -0.33, P < 0.001	rho = -0.37, P < 0.001	rho = 0.11, P = 0.142

Multifactorial analysis of the study scales

A multifactorial linear regression was applied with the dependent variable being the overall score of the MICA-4 and independent variables being the demographic characteristics of the participants, questions related to perinatal mental disorders, the score on how midwives perceive different groups of pregnant women, the percentage score on perinatal mental health knowledge, the overall score of the PMHA, and the “Emotion” and “Education” dimensions of the PIMMHS. The results of the analysis are described in Table 13.

**Table 13:** Multifactorial regression analysis with the dependent variable being the overall score of the Mental Illness Clinicians' Attitudes Scale. β, regression coefficient; SE, standard error of the coefficient; PIMMHS, Professional Issues in Maternal Mental Health Scale; PMHA, Perinatal Mental Health Awareness

Variable	β	SE	P
Age			
20-30 (reference)	-	-	-
31-40	-0.03	0.04	0.498
40+	0.001	0.06	0.978
Years worked as a midwife			
Less than 5 (reference)	-	-	-
6-15	0.03	0.05	0.577
16+	0.01	0.06	0.858
How often do you care for a pregnant woman with mental illness?			
Never (reference)	-	-	-
Rarely	0.05	0.04	0.193
Sometimes/often	0.001	0.04	0.980
Sector of midwifery work			
Prenatal services (reference)	-	-	-
Perinatal services	0.01	0.05	0.840
Postnatal services	-0.03	0.05	0.580
All sectors	-0.07	0.04	0.094
Experience in mental health			
No (reference)	-	-	-
Yes	-0.04	0.05	0.471
Participation in perinatal mental health training			
No (reference)			
Yes	-0.03	0.03	0.262
Feeling well-trained to support women with perinatal mental disorders			
No (reference)	-	-	-
Yes	-0.05	0.04	0.248
Access to information about perinatal mental disorders			
No (reference)	-	-	-
Yes	-0.02	0.03	0.431
Perception of different groups of pregnant women	0.003	0.001	0.023
Percentage score of perinatal mental health knowledge	-0.004	0.001	<0.001
Overall PMHA score	0.001	0.004	0.852
PIMMHS - Emotion	-0.01	0.01	0.008
PIMMHS - Education	0.0004	0.01	0.956

The perception score of how midwives view different groups of pregnant women, the percentage score of participants' perinatal mental health knowledge, and the “Emotion” dimension of the PIMMHS scale were independently associated with the overall score of the MICA-4 scale. More specifically, a higher score on how midwives perceive different groups of pregnant women appears to lead to an increase in the MICA-4 score, indicating more stigma-related attitudes toward mental illnesses and psychiatry (β=0.003, SE=0.001, p=0.023). An increase in the percentage score of perinatal mental health knowledge is associated with a decrease in the MICA-4 score, reflecting less stigma-related attitudes toward mental illnesses and psychiatry (β=-0.004, SE=0.001, p<0.001). A higher score on the “Emotion” dimension of the PIMMHS scale leads to a decrease in the MICA-4 score, indicating less stigma-related attitudes toward mental illnesses and psychiatry (β=-0.01, SE=0.01, p=0.008).

Subsequently, a multivariate linear regression was applied with the dependent variable being the participants' attitude scores toward perinatal mental health and independent variables being participants' demographic data, questions related to perinatal mental health disorders, the score on how midwives perceive different groups of pregnant women, the percentage score of perinatal mental health knowledge, the overall score of the PMHA, and the "Emotion" and "Education" dimensions of the PIMMHS. The results of the analysis are presented in Table 14.

**Table 14:** Multifactorial regression analysis with the dependent variable being the attitudes score of participants for perinatal mental health. Note: the analysis was conducted using logarithmic transformations. β, Regression coefficient; SE, standard error of the coefficient; PIMMHS, Professional Issues in Maternal Mental Health Scale; PMHA: Perinatal Mental Health Awareness

Variable	β	SE	P
Age			
20-30 (reference)	-	-	-
31-40	-0.06	0.02	0.012
40+	-0.06	0.03	0.030
Years worked as a midwife			
Less than 5 (reference)	-	-	-
6-15	0.04	0.02	0.061
16+	0.04	0.03	0.180
How often do you care for a pregnant woman with mental illness?			
Never (reference)	-	-	-
Rarely	-0.002	0.02	0.921
Sometimes/often	0.01	0.02	0.786
Sector of midwifery work			
Prenatal services (reference)	-	-	-
Perinatal services	-0.02	0.03	0.389
Postnatal services	-0.01	0.03	0.605
All sectors	-0.01	0.02	0.541
Experience in mental health			
No (reference)	-	-	-
Yes	0.003	0.03	0.907
Participation in perinatal mental health training			
No (reference)	-	-	-
Yes	-0.02	0.01	0.279
Feeling well-trained to support women with perinatal mental disorders			
No (reference)	-	-	-
Yes	0.01	0.02	0.638
Access to information about perinatal mental disorders			
No (reference)	-	-	-
Yes	0.01	0.02	0.720
Perception of different groups of pregnant women	0.0002	0.001	0.748
Percentage score of perinatal mental health knowledge	0.004	0.004	<0.001
Overall PMHA score	0.004	0.002	0.028
PIMMHS - Emotion	0.002	0.003	0.556
PIMMHS - Education	0.004	0.004	0.351

The age of the participants, the percentage score of perinatal mental health knowledge, and the overall PMHA were independently found to correlate with the participants' attitude scores toward perinatal mental health. More specifically, participants aged 31 to 40 years or over 40 years had lower attitude scores toward perinatal mental health, indicating a less positive attitude toward pregnant women with mental health issues compared to participants aged 20 to 30 years (β=-0.06, SE=0.02, p=0.012 and β=-0.06, SE=0.03, p=0.030, respectively).

An increase in the percentage score of knowledge about perinatal mental health is associated with an increase in attitude scores, indicating a more positive attitude toward pregnant women with mental health issues (β=0.004, SE=0.004, p<0.001). In addition, a higher score on the PMHA leads to a more positive attitude among participants toward pregnant women with mental health issues (β=0.004, SE=0.002, p=0.028).

A multifactorial linear regression was applied with the dependent variable being the overall score of the PMHA and independent variables being the demographic characteristics of the participants, questions related to perinatal mental disorders, the score on how midwives perceive different groups of pregnant women, the percentage score on perinatal mental health knowledge, and the “Emotion” and “Education” dimensions of the PIMMHS. The results of the analysis are described in Table 15.

**Table 15:** Multifactorial regression analysis with the dependent variable being the overall score of the Perinatal Mental Health Awareness Scale Note: the analysis was conducted using logarithmic transformations β, regression coefficient; SE, standard error of the coefficient; PIMMHS, Professional Issues in Maternal Mental Health Scale

Variable	β	SE	P
Age			
20-30 (reference)	-	-	-
31-40	-0.11	0.06	0.049
40+	-0.05	0.08	0.497
Years worked as a midwife			
Less than 5 (reference)	-	-	-
6-15	0.05	0.06	0.396
16+	0.02	0.07	0.814
How often do you care for a pregnant woman with mental illness?			
Never (reference)	-	-	-
Rarely	0.06	0.05	0.220
Sometimes/often	0.17	0.05	0.001
Sector of midwifery work			
Prenatal services (reference)	-	-	-
Perinatal services	0.03	0.07	0.712
Postnatal services	0.03	0.07	0.681
All sectors	0.02	0.05	0.683
Experience in mental health			
No (reference)	-	-	-
Yes	0.06	0.07	0.398
Participation in perinatal mental health training			
No (reference)	-	-	-
Yes	0.01	0.04	0.792
Feeling well-trained to support women with perinatal mental disorders			
No (reference)	-	-	-
Yes	0.12	0.05	0.024
Access to information about perinatal mental disorders			
No (reference)	-	-	-
Yes	0.07	0.04	0.109
Perception of different groups of pregnant women	-0.001	0.002	0.630
PIMMHS - Emotion	0.01	0.01	0.195
PIMMHS - Education	0.01	0.01	0.232

The age of the participants, how often they cared for a pregnant woman with mental illness, and whether they felt well-trained to support women with perinatal mental disorders were found to independently correlate with the overall score of the PMHA. More specifically, participants aged 31 to 40 years had a lower score on the PMHA scale, indicating less knowledge about managing and organizing situations related to perinatal mental health compared to participants aged 20 to 30 years (β=-0.11, SE=0.06, p=0.049).

Participants who sometimes or often cared for a pregnant woman with mental illness had a higher score on the PMHA scale, indicating better knowledge of managing and organizing situations related to perinatal mental health compared to those who never cared for a pregnant woman with mental illness (β=0.17, SE=0.05, p=0.001).

Participants who felt well-trained to support women with perinatal mental disorders had better knowledge about managing and organizing situations related to perinatal mental health compared to those who did not feel well-trained (β=0.12, SE=0.05, p=0.024).

A multifactorial linear regression was applied with the dependent variable being the percentage knowledge score of perinatal mental health and independent variables being the demographic characteristics of the participants and questions related to perinatal mental disorders. The results of the analysis are described in Table 16.

**Table 16:** Multifactorial regression analysis with the dependent variable being the percentage knowledge score of perinatal mental health. Note: the analysis was conducted using logarithmic transformations. β, regression coefficient; SE, standard error of the coefficient

Variable	β	SE	P
Age			
20-30 (reference)	-	-	-
31-40	-0.14	0.08	0.096
40+	-0.28	0.11	0.011
Years worked as a midwife			
Less than 5 (reference)	-	-	-
6-15	0.16	0.09	0.064
16+	0.30	0.11	0.006
How often do you care for a pregnant woman with mental illness?			
Never (reference)	-	-	-
Rarely	0.14	0.07	0.042
Sometimes/often	0.18	0.07	0.019
Sector of midwifery work			
Prenatal services (reference)	-	-	-
Perinatal services	-0.05	0.10	0.646
Postnatal services	0.01	0.11	0.931
All sectors	0.10	0.08	0.219
Experience in mental health			
No (reference)	-	-	-
Yes	-0.11	0.10	0.252
Participation in perinatal mental health training			
No (reference)	-	-	-
Yes	0.04	0.05	0.508
Feeling well-trained to support women with perinatal mental disorders			
No (reference)	-	-	-
Yes	0.10	0.07	0.164
Access to information about perinatal mental disorders			
No (reference)	-	-	-
Yes	0.05	0.06	0.427

The age of the participants, the years they had worked as a midwife, and how often they cared for a pregnant woman with a mental illness were found to be independently associated with the percentage knowledge score of perinatal mental health. More specifically, participants over 40 years old had lower percentage knowledge scores of perinatal mental health, indicating less knowledge compared to participants aged 20 to 30 years (β=-0.28, SE=0.11, p=0.011).

Participants who had worked as midwives for more than 16 years had higher percentage knowledge scores of perinatal mental health, indicating better knowledge compared to those who had worked for less than 5 years (β=0.30, SE=0.11, p=0.006).

Participants who rarely, sometimes, or often cared for pregnant women with mental illness had higher percentage knowledge scores compared to those who had never cared for such women (β=0.14, SE=0.07, p=0.042 and β=0.18, SE=0.07, p=0.019, respectively).

## Discussion

The perinatal period is recognized as a significant transitional phase that brings about changes in a woman’s relationships with her partner, family, friends, and broader social network [26]. As part of this transition, women may also experience emotional shifts ranging from mild discomfort and anxiety to severe psychological distress. The role of midwives is particularly crucial in providing mental health services during the perinatal period. This highlights the importance of their personal attitudes and perceptions, as these can influence their professional practice [27]. Midwives report an interest in providing mental health care and acknowledge their integral role in delivering perinatal mental health services [17,28-31].

The research data showed that midwives' knowledge of mental health affects their attitudes towards it. The midwives in the sample reported having the knowledge and ability to manage specific mental health issues such as depression. This finding contrasts with other studies that indicate a lack of knowledge and skills regarding women’s perinatal mental health issues [16,17,32] and a lack of confidence in adequately assessing and managing perinatal mental health problems [11]. Specifically, gaps have been identified in certain areas such as risk factors and available treatment options. Furthermore, several studies have found that prenatal depression is less recognized by midwives compared to postpartum depression [16,32]. Regarding mental health conditions such as postpartum psychosis, schizophrenia, bipolar affective disorder, and personality disorders, midwives' knowledge is particularly low [16,17]. Notably, Jones et al. [28] found that midwives had difficulty recognizing prenatal and postpartum depression, emphasizing the need for further education to ensure their ability to conduct psychosocial assessments and manage these women. Similarly, Afolayan et al. [18] identified a knowledge deficit among midwives concerning the risk factors and detection methods of postpartum depression, recommending the development of periodic training programs. In the present study, midwives felt confident in managing such issues but expressed difficulties in addressing others, such as learning disabilities.

Midwives’ knowledge was also correlated with their attitudes toward pregnant women with mental health problems in this study. Those with greater knowledge did not exhibit stigmatizing attitudes, while those with less knowledge tended to hold more negative views. Studies evaluating midwives' attitudes and behaviors conclude that they generally hold positive attitudes toward mental health issues and their management and that their lack of skills stems from insufficient training rather than a lack of interest or willingness [17,27]. Findings from studies involving midwives indicate that many do not feel comfortable providing perinatal mental health services because they lack the necessary training and, consequently, do not feel confident in doing so. Additionally, some midwives do not feel comfortable due to personal attitudes and fears of encountering problems. Nonetheless, they acknowledge the need for training in this area [17,28]. The fear or lack of confidence among midwives does not imply an unwillingness to provide perinatal mental health services, as they appear to understand the importance of such services [28]. However, the negative attitude of some midwives toward women with mental health issues should not be overlooked. Being part of the healthcare system does not necessarily mean they possess comprehensive knowledge of mental health, which can lead to the formation of negative attitudes toward these women, both as individuals and in their roles as mothers. Given the close interaction midwives have with these women, it is understandable that if the latter perceive the former as having a negative attitude toward them, the outcomes will not be positive. Interactions marked by negative attitudes not only fail to lead to interventions that support these women but may also make them hesitant to approach midwives for help [3].

The need for education was emphasized by the majority of midwives in this study. The data revealed that further training and education are required to enhance midwives' skills, knowledge, and confidence in providing perinatal mental health care [33,34]. Studies assessing the effectiveness of educational interventions in improving midwives' knowledge and skills have found that with appropriate training, midwives can build the necessary competencies and confidence to provide perinatal mental health care [35-37]. A study conducted by McLachlan et al. [36] evaluated communication skills training for midwives and found that it increased their self-reported comfort and ability to identify and care for women with psychosocial problems during the postpartum period. Similarly, the study conducted by Reed et al. [37], which examined midwives' experiences in acquiring new counseling skills and providing counseling interventions to women who had experienced traumatic childbirth, revealed that midwives were able to learn and apply advanced counseling skills, thus gaining confidence in providing mental health support. The study by Fenwick et al. [35] evaluated the outcomes of a counseling intervention training for midwives and found that the training significantly improved midwives' knowledge, skills, and confidence in counseling women on psychosocial issues.

These findings underscore the need for the development and evaluation of training programs for midwives. Given the close contact midwives have with women during the perinatal period, they should be capable of not only educating women about mental health issues but also identifying those at risk of developing mental health disorders so that they can refer them to specialists for timely intervention and support. Several studies have highlighted that midwives play a crucial role in the assessment and management of perinatal mental health [16,17]. In providing woman-centered holistic care, midwives must consider the importance of women’s psychological well-being [38] and the psychosocial risks women face during the prenatal period [39].

This study has several strengths. It used validated psychometric tools, such as the PIMMHS, the MICA-4, and the PMHA scale, enabling a comprehensive assessment of midwives’ knowledge, attitudes, and self-reported competence regarding perinatal mental health. The inclusion of a large and geographically diverse sample of 223 midwives from urban, rural, and island regions across Greece enhances the generalizability of the findings within the national context. Additionally, the study addresses a critical gap in the literature by exploring midwives' educational needs in perinatal mental health, a subject that has received limited attention in Greece. The findings provide actionable insights for designing targeted training programs to improve midwifery care in this area.

However, there are limitations to this study. The reliance on self-reported measures to assess knowledge and perceived competence introduces the possibility of response bias, as participants may have over- or under-estimated their abilities or attitudes. Furthermore, the study used indirect methods to evaluate skills rather than direct observation or practical assessments, which would have provided a more accurate evaluation of midwives’ capabilities in managing perinatal mental health issues. The use of convenience and snowball sampling methods may have introduced selection bias, limiting the representativeness of the sample. Additionally, the cross-sectional design captures data at a single point in time, preventing an assessment of changes over time or the long-term impact of interventions. Lastly, as the study focused on Greece, its findings may have limited applicability to other healthcare systems or cultural contexts.

## Conclusions

This study highlights significant gaps in the knowledge and confidence of midwives regarding perinatal mental health. Despite recognizing their role in supporting women with mental health issues, many midwives feel inadequately trained to address conditions such as bipolar disorder, schizophrenia, and anxiety. The findings suggest that improved education and training are necessary to bridge these gaps, with many midwives expressing a strong desire for further professional development in these areas. By addressing these training needs, midwives can play a more effective role in identifying and managing perinatal mental health issues, ultimately improving outcomes for mothers and their infants. The results underscore the importance of integrating mental health education into midwifery training programs and ensuring ongoing professional development opportunities to meet the growing demand for mental health services in maternity care.

## Appendices

Appendix A

Questionnaire

Pre-psychometric testing version of the Professional Issues in Maternal Mental Health Scale (PIMMHS) is displayed in Table 17.

**Table 17:** Pre-psychometric testing version of the Professional Issues in Maternal Mental Health Scale.

Professional Issues in Perinatal Mental Health	Strongly agree	Agree	Neither agree nor disagree	Disagree	Strongly disagree
Q1. I know exactly who to contact if a woman is experiencing mental health problems	•	•	•	•	•
Q2. Sometimes I feel reluctant to discuss emotional problems that a woman might be having as I feel uncomfortable discussing these with her	•	•	•	•	•
Q3. Training pays sufficient attention to the cultural dimensions of pregnancy, birth, and postnatal care	•	•	•	•	•
Q4. It is easy for me to obtain help for women with mental health problems	•	•	•	•	•
Q5. Sometimes I feel reluctant to discuss emotional problems that a woman might be having as I know I am not going to have enough time to deal with them	•	•	•	•	•
Q6. Sometimes I feel reluctant to discuss emotional problems that a woman might be having because I would not know what to do or who to ask for advice	•	•	•	•	•
Q7. There are some emotional issues that women should really not discuss with midwives, are too private, and should be discussed with her partner	•	•	•	•	•
Q8. It is difficult to discuss mental health problems with women in the antenatal clinic	•	•	•	•	•
Q9. Antenatal clinics are not the best place to discuss a woman’s mental health problems	•	•	•	•	•
Q10. Sometimes I feel reluctant to discuss emotional problems that a woman may be experiencing as I don’t feel adequately trained to deal with these issues	•	•	•	•	•

Post-psychometric testing version of the PIMMHS is given in Table 18.

**Table 18:** Post-psychometric testing version of PIMMHS PIMMHS, Professional Issues in Maternal Mental Health Scale

PIMMHS - Emotion	Strongly agree	Agree	Neither agree nor disagree	Disagree	Strongly disagree
Q1. I know exactly who to contact if a woman is experiencing mental health problems	•	•	•	•	•
Q2. Sometimes I feel reluctant to discuss emotional problems that a woman might be having as I feel uncomfortable discussing these with her	•	•	•	•	•
Q3. Sometimes I feel reluctant to discuss emotional problems that a woman might be having as I know I am not going to have enough time to deal with them	•	•	•	•	•
Q4. Sometimes I feel reluctant to discuss emotional problems that a woman might be having because I would not know what to do or who to ask for advice	•	•	•	•	•
PIMMHS - Training	Strongly agree	Agree	Neither agree nor disagree	Disagree	Strongly disagree
Q1. Sometimes I feel reluctant to discuss emotional problems that a woman may be experiencing as I don’t feel adequately trained to deal with these issues	•	•	•	•	•
Q2. Training pays sufficient attention to the cultural dimensions of pregnancy, birth, and postnatal care	•	•	•	•	•
Q3. It is easy for me to obtain help for women with mental health problems	•	•	•	•	•

Mental Illness Clinicians’ Attitudes Scale (MICA-2)

Instructions

For each of the questions 1-16, please respond by ticking one box only. Mental illness here refers to conditions for which an individual would be seen by a psychiatrist (Table 19).

**Table 19:** Mental Illness Clinicians’ Attitudes Scale (MICA-2).

Question	Strongly agree	Agree	Somewhat agree	Somewhat disagree	Disagree	Strongly disagree
1. I just learn about mental health when I have to, and would not bother reading additional material on it.	-	-	-	-	-	-
2. People with a severe mental illness can never recover enough to have a good quality of life.	-	-	-	-	-	-
3. Working in the mental health field is just as respectable as other fields of health and social care.	-	-	-	-	-	-
4. If I had a mental illness, I would never admit this to my friends because I would fear being treated differently.	-	-	-	-	-	-
5. People with a severe mental illness are dangerous more often than not.	-	-	-	-	-	-
6. Health/social care staff know more about the lives of people treated for a mental illness than do family members or friends.	-	-	-	-	-	-
7. If I had a mental illness, I would never admit this to my colleagues for fear of being treated differently.	-	-	-	-	-	-
8. Being a health/social care professional in the area of mental health is not like being a real health/social care professional.	-	-	-	-	-	-
9. If a senior colleague instructed me to treat people with a mental illness in a disrespectful manner, I would not follow their instructions.	-	-	-	-	-	-
10. I feel just as comfortable talking to someone with a mental illness as I do speaking with someone who has a physical illness.	-	-	-	-	-	-
11. It is important that any health/social care professional supporting a person with a mental illness also ensures that their physical health is assessed	-	-	-	-	-	-
12. The public does not need protection from individuals with severe mental illness	-	-	-	-	-	-
13. If a person with a mental illness complained of physical symptoms (such as chest pain) I would attribute it to their mental illness.	-	-	-	-	-	-
14. General practitioners should not be expected to complete a thorough assessment for people with psychiatric symptoms because they can be referred to a psychiatrist.	-	-	-	-	-	-
15. I would use the terms “crazy,” “nutter,” “mad,” etc. to describe to colleagues people with a mental illness who I have seen in my work.	-	-	-	-	-	-
16. If a colleague told me they had a mental illness, I would still want to work with them	-	-	-	-	-	-

Perinatal Mental Health Awareness (PMHA) scale is displayed in Table 20.

**Table 20:** Perinatal Mental Health Awareness scale HIV, human immunodeficiency virus

PMHA SAD: Question 1				
How knowledgeable you are about the following conditions:				
Condition	Very knowledgeable	Knowledgeable	Not very knowledgeable	Not knowledgeable
Stress, anxiety, and depression	-	-	-	-
Learning disabilities	-	-	-	-
Medical, obstetric conditions (e.g., pre-eclampsia, HIV, symphysis pubis dysfunction)	-	-	-	-
PMHA: LD - Question 2				
How confident are you in identifying the following conditions:				
Condition	Very knowledgeable	Knowledgeable	Not very knowledgeable	Not knowledgeable
Stress, anxiety, and depression	-	-	-	-
Learning disabilities	-	-	-	-
Medical, obstetric conditions (e.g., pre-eclampsia, HIV, symphysis pubis dysfunction)	-	-	-	-
PMHA: MED - Question 3				
How confident are you in managing the following conditions:				
Condition	Very knowledgeable	Knowledgeable	Not very knowledgeable	Not knowledgeable
Stress, anxiety, and depression	-	-	-	-
Learning disabilities	-	-	-	-
Medical, obstetric conditions (e.g., pre-eclampsia, HIV, symphysis pubis dysfunction)	-	-	-	-

Your Learning Needs for Professional Development

I feel well trained to support women with perinatal mental health disorders.

□No □Yes

I feel that I have sufficient access to information regarding mental health disorders during pregnancy and the perinatal period.

□No □Yes

For which of the following mental health disorders would you like further training? (check all that apply):

□Bipolar disorder □Schizophrenia □Personality disorders □Anxiety disorders □

Tokophobia □Substance-related disorders □Depression

□Other (please describe):

□I do not need further training

In which of the following areas of knowledge would you like further training? (check all that apply):

□Signs and symptoms of a mental illness □Roles of other healthcare professionals □Understanding care options □Child safety □Impact of childbirth on mental health □Other (please describe):

□I do not need further training

In which of the following skills would you like further training? (check all that apply):

□Mental health assessment □Communication skills □Risk assessment for developing a mental illness

□Managing anxiety and aggression □Clinical management (medication, etc.)

□Working with families and caregivers □Support for breastfeeding □Discharge planning from the hospital

Other (please describe):

□I do not need further training

What type of training would you prefer? (check all that apply):

□A series of workshops and seminars □Access to online modules □Seminar with a guest speaker: half-day or full-day (one cycle) □Workshop (with active participation): half-day or full-day (one cycle) □Other:

Your Opinions - Please complete this questionnaire without discussing its content with others. It is important to know your opinions and learning needs in order to improve professional development opportunities.

(Circle your desired response) 1 = Strongly Disagree, 2 = Disagree, 3 = Neither Agree nor Disagree, 4 = Agree, 5 = Strongly Agree)

Questionnaire regarding the opinions is displayed in Table 21.

**Table 21:** Your opinions.

Your Opinions	1	2	3	4	5
Attitude: It is easy to recognize mental health issues in pregnant women.	1	2	3	4	5
Knowledge: Hormones released during pregnancy protect against mental illness.	1	2	3	4	5
Knowledge: Pregnant women should not take psychiatric medications.	1	2	3	4	5
Knowledge: Women should not breastfeed if they are taking medication for mental illness.	1	2	3	4	5
Attitude: Pregnant women with mental health issues should always be referred to a specialist.	1	2	3	4	5
Knowledge: The cutoff score for further assessment on the EPDS (Edinburgh Postnatal Depression Scale) is 9.	1	2	3	4	5
Attitude: Pregnant women with mental illness are likely to be difficult to manage.	1	2	3	4	5
Knowledge: Women with mental illness may have attachment issues with their newborn.	1	2	3	4	5
Attitude: It is unlikely that women with mental illness will recover.	1	2	3	4	5
Knowledge: A mental illness during pregnancy can affect the newborn's birth weight.	1	2	3	4	5
Attitude: It is not the midwife's role to assess the mental health of a woman in her care.	1	2	3	4	5
Knowledge: Family history of mental illness does not pose a risk for mental illness during pregnancy.	1	2	3	4	5
Attitude: Women with severe mental illness should not be encouraged to have children.	1	2	3	4	5
Knowledge: The EPDS (Edinburgh Postnatal Depression Scale) is a useful diagnostic tool for depression and anxiety.	1	2	3	4	5
Knowledge: Women with postpartum depression are sad and cry all the time.	1	2	3	4	5
Knowledge: A previous history of mental illness is a risk factor for developing mental illness during pregnancy.	1	2	3	4	5

Your Knowledge

It is important to understand your learning needs, so please complete this section without discussing the scenarios with others.

Scenario 1: You are working in a home maternity care service and are doing a home follow-up with Anna, a first-time mother whose newborn is less than a week old. Anna says she is not sleeping. She is terrified of sudden infant death syndrome (SIDS) and describes staying awake to watch her baby in case they stop breathing. She feels very nervous about handling her baby, as she is afraid she will drop them. She is also worried about breastfeeding, as she can't see how much milk the baby is getting and believes something may be wrong with her baby. Anna’s partner has arranged for his mother to stay with her as he has returned to work. Anna is due to see her doctor in the morning as she feels something is wrong with the baby and has also booked an appointment to see a chiropractor, believing they will “adjust” her baby. After your assessment, the baby is afebrile and has gained 30 grams since yesterday. Before pregnancy, Anna worked as an accountant, and her husband describes her as obsessive about organization.

Is there something wrong with the person in the scenario above?

Simple  answer:  ……………………………………………………………………………………………...

Scenario 2: Su-lin is 24 years old and is pregnant without having planned it. She lives with her parents, and despite enrolling in university (pharmacy), she has failed several courses and dropped out in the past year. She has always been shy, but over the last six months, she has stopped seeing her friends and spends much time alone in her room. She no longer takes care of her personal hygiene. Her parents hear Su-lin walking around her bedroom at night, and although they know she is alone, they have heard her arguing and yelling as if someone else is there. When they try to encourage her to do more, she tells them she won't leave the house because she believes the neighbors are spying on her. She has also restricted her diet, worrying that the food may be contaminated by radiation from nearby cell phone towers. There is no history of drug use. When her pregnancy was confirmed at 12 weeks, Su-lin refused any prenatal care. Is there something wrong with the person in the scenario above?

Simple answer:

…………………………………………………………………………………………………..

Scenario 3: Isabella is a 28-year-old multigravida visiting the maternity clinic at 28 weeks of pregnancy. Over the past few weeks, she has been feeling unusually sad and miserable. She has lost interest in most of her daily activities and complains of a loss of energy and insomnia on a daily basis. Isabella says she feels worthless and expresses hopelessness about the future. Her husband is often away due to his work, and Isabella feels unsupported even when he is around. She comments that daily activities seem overwhelmingly difficult and she has been very irritable with her 5-year-old daughter.

She is also more distracted and has neglected household chores.

Is there something wrong with the person in the scenario above?

Simple  answer:  ……………………………………………………………………………………………

Scenario 4: Sally, a 30-year-old first-time mother, is a professional who has been happily married to Tom for five years. She was well throughout her pregnancy, but three days after giving birth, the staff noticed that Sally wasn’t sleeping well, only sleeping 20 minutes at a time. Despite the lack of sleep, she describes feeling full of energy and is making lots of plans for her baby and home renovations. Although she has no history of drug use, she feels euphoric about the birth and her new son, showing excitement to all the staff and visitors. The staff have raised concerns about safety due to the way she is energetically carrying the baby around the work area continuously, but Sally doesn’t seem worried. She has also been seen laughing to herself frequently, and her husband is concerned that she isn’t acting like her usual self.

Is there something wrong with the person in the scenario above?

Simple  answer:  …………………………………………………………………………………………..

Your Impression of Other Midwives' Opinions

The following questions focus on how midwives perceive different groups of pregnant women. We are interested in how you think OTHER MIDWIVES GENERALLY see these women.

The questionnaire for the impression of other midwives' opininios is displayed in Table 22.

We are not asking about your personal views on these groups, but how you believe MOST MIDWIVES view them.

**Table 22:** Your impression of other midwives' opinions

As most midwives see it...	Not at all…………………………….....Very much				
-	1	2	3	4	5
… how capable and skillful are pregnant women who also manage a household?	1	2	3	4	5
… how warm and friendly are pregnant women who also manage a household?	1	2	3	4	5
… how capable and skillful are pregnant women who have a physical disability?	1	2	3	4	5
… how warm and friendly are pregnant women who have a physical disability?	1	2	3	4	5
… how capable and skillful are pregnant women who have a substance addiction?	1	2	3	4	5
… how warm and friendly are pregnant women who have a substance addiction?	1	2	3	4	5
… how capable and skillful are pregnant women who run their own business?	1	2	3	4	5
… how capable and skillful are pregnant women who have bipolar disorder?	1	2	3	4	5
… how warm and friendly are pregnant women who have bipolar disorder?	1	2	3	4	5
… how capable and skillful are pregnant women who have anxiety?	1	2	3	4	5
… how warm and friendly are pregnant women who have anxiety?	1	2	3	4	5
… how capable and skillful are pregnant women who have housing issues?	1	2	3	4	5
… how warm and friendly are pregnant women who have housing issues?	1	2	3	4	5
… how capable and skillful are pregnant women who have depression?	1	2	3	4	5
… how warm and friendly are pregnant women who have depression?	1	2	3	4	5
… how capable and skillful are pregnant women who have schizophrenia?	1	2	3	4	5
… how warm and friendly are pregnant women who have schizophrenia?	1	2	3	4	5

About You

Your age:

□20-25 □26-30 □31-35 □36-40 □41-45 □46-50 □51-55 □55-60 □60+

How many years have you worked as a midwife?

□Less than a year □1-2 □3-5 □6-10 □11-15 □16-20 □21-25 □26-30 □30+

How often do you care for a pregnant woman with a mental illness?

□Never □Rarely □Sometimes □Often □I'm not sure

In which area of midwifery are you currently working?

□Prenatal services □Perinatal services □Postnatal services □I work in all these areas

Do you have work experience in mental health services?

□No □Yes

Have you participated in any training (seminar, workshop, online) in the last two years that improved your knowledge and skills in perinatal mental health?

□No □Yes

Any comments you would like to add about this survey:

## References

[1] Michalczyk J, Miłosz A, Soroka E (2023). Postpartum psychosis: a review of risk factors, clinical picture, management, prevention, and psychosocial determinants. Med Sci Monit.

[2] Friedman SH, Reed E, Ross NE (2023). Postpartum psychosis. Curr Psychiatry Rep.

[3] Viguera AC, Whitfield T, Baldessarini RJ (2007). Risk of recurrence in women with bipolar disorder during pregnancy: prospective study of mood stabilizer discontinuation. Am J Psychiatry.

[4] Agrawal I, Mehendale AM, Malhotra R (2022). Risk factors of postpartum depression. Cureus.

[5] Glasser S, Hadad L, Bina R, Boyko V, Magnezi R (2016). Rate, risk factors and assessment of a counselling intervention for antenatal depression by public health nurses in an Israeli ultra-orthodox community. J Adv Nurs.

[6] Luoma I, Korhonen M, Salmelin RK, Helminen M, Tamminen T (2015). Long-term trajectories of maternal depressive symptoms and their antenatal predictors. J Affect Disord.

[7] Watson HJ, Von Holle A, Hamer RM (2013). Remission, continuation and incidence of eating disorders during early pregnancy: a validation study in a population-based birth cohort. Psychol Med.

[8] Barber CC (2009). Perinatal mental health care in New Zealand: the promise of beginnings. N Z J Psychol.

[9] Challacombe FL, Wroe AL (2013). A hidden problem: consequences of the misdiagnosis of perinatal obsessive-compulsive disorder. Br J Gen Pract.

[10] Taillieu TL, Brownridge D (2010). Violence against pregnant women: Prevalence, patterns, risk factors, theories, and directions for future research. Aggress Violent Behav.

[11] McCauley K, Elsom S, Muir-Cochrane E, Lyneham J (2011). Midwives and assessment of perinatal mental health. J Psychiatr Ment Health Nurs.

[12] Bodén R, Lundgren M, Brandt L, Reutfors J, Andersen M, Kieler H (2012). Risks of adverse pregnancy and birth outcomes in women treated or not treated with mood stabilisers for bipolar disorder: population based cohort study. BMJ.

[13] Ding XX, Wu YL, Xu SJ (2014). Maternal anxiety during pregnancy and adverse birth outcomes: a systematic review and meta-analysis of prospective cohort studies. J Affect Disord.

[14] Özerdem A, Akdeniz F (2014). Pregnancy and postpartum in bipolar disorder. Neuropsychiatry.

[15] Butler MM, Sheehy L, Kington MM (2015). Evaluating midwife-led antenatal care: choice, experience, effectiveness, and preparation for pregnancy. Midwifery.

[16] Ross-Davie M, Elliott S, Green L (2007). Planning and implementing mental health training. Br J Midwifery.

[17] Hauck YL, Kelly G, Dragovic M, Butt J, Whittaker P, Badcock JC (2015). Australian midwives knowledge, attitude and perceived learning needs around perinatal mental health. Midwifery.

[18] Afolayan JA, Onasoga OA, Rejuaro FM, Yusuf ARG, Onuabueke C (2016). Knowledge of postpartum depression and its associated risk factors among nurse-midwives in a Nigerian tertiary hospital. Sierra Leone J Biomed Res.

[19] Coates D, Foureur M (2019). The role and competence of midwives in supporting women with mental health concerns during the perinatal period: a scoping review. Health Soc Care Community.

[20] Magdalena CD, Tamara WK (2020). Antenatal and postnatal depression - are Polish midwives really ready for them?. Midwifery.

[21] Chatzigeorgiou E (2021). The impact of the COVID-19 pandemic on perinatal mental health. 28th Pancyprian Nursing and Midwifery Conference Proceedings.

[22] Pagagiannaki A (2021). Investigating the Experiences of New and Pregnant Mothers Regarding Perinatal Care Services Provided in Public Institutions in Crete During the COVID-19 Pandemic. Investigating the experiences of new and pregnant mothers regarding perinatal care services provided in public institutions in Crete during the COVID-19 pandemic.

[23] Jomeen J, Jarrett P, Martin CR (2018). Professional issues in maternal mental health scale (PIMMHS): the development and initial validation of a brief and valid measure. Eur J Midwifery.

[24] Gabbidon J, Clement S, van Nieuwenhuizen A, Kassam A, Brohan E, Norman I, Thornicroft G (2013). Mental Illness: Clinicians' Attitudes (MICA) scale-psychometric properties of a version for healthcare students and professionals. Psychiatry Res.

[25] Martin CR, Jomeen J, Jarrett P (2017). The development and initial validation of the perinatal mental health awareness scale in student midwives. J Midwifery Reprod Health.

[26] Jonsdottir SS, Thome M, Steingrimsdottir T, Lydsdottir LB, Sigurdsson JF, Olafsdottir H, Swahnberg K (2017). Partner relationship, social support and perinatal distress among pregnant Icelandic women. Women Birth.

[27] Noonan M, Jomeen J, Galvin R, Doody O (2018). Survey of midwives' perinatal mental health knowledge, confidence, attitudes and learning needs. Women Birth.

[28] Jones CJ, Creedy DK, Gamble JA (2012). Australian midwives' awareness and management of antenatal and postpartum depression. Women Birth.

[29] Mathibe-Neke J, Rothberg A, Langley G (2014). The perception of midwives regarding psychosocial risk assessment during antenatal care. Health SA Gesondheid.

[30] Noonan M, Galvin R, Doody O, Jomeen J (2017). A qualitative meta-synthesis: public health nurses role in the identification and management of perinatal mental health problems. J Adv Nurs.

[31] Ross-Davie M, Elliott S, Sarkar A, Green G (2006). A public health role in perinatal mental health: are midwives ready?. Br J Midwifery.

[32] Jones CJ, Creedy DK, Gamble JA (2011). Australian midwives' knowledge of antenatal and postpartum depression: a national survey. J Midwifery Womens Health.

[33] Higgins A, Downes C, Monahan M, Gill A, Lamb SA, Carroll M (2018). Barriers to midwives and nurses addressing mental health issues with women during the perinatal period: The Mind Mothers study. J Clin Nurs.

[34] Lau R, McCauley K, Barnfield J, Moss C, Cross W (2015). Attitudes of midwives and maternal child health nurses towards suicide: a cross-sectional study. Int J Ment Health Nurs.

[35] Fenwick J, Toohill J, Slavin V, Creedy DK, Gamble J (2018). Improving psychoeducation for women fearful of childbirth: evaluation of a research translation project. Women Birth.

[36] McLachlan HL, Forster DA, Collins R, Gunn J, Hegarty K (2011). Identifying and supporting women with psychosocial issues during the postnatal period: evaluating an educational intervention for midwives using a before-and-after survey. Midwifery.

[37] Reed M, Fenwick J, Hauck Y, Gamble J, Creedy DK (2014). Australian midwives' experience of delivering a counselling intervention for women reporting a traumatic birth. Midwifery.

[38] Elliott S, Ross-Davie M, Sarkar A, Green L (2007). Detection and initial assessment of mental disorder: the midwife's role. Br J Midwifery.

[39] Williams CJ, Turner KM, Burns A, Evans J, Bennert K (2016). Midwives and women's views on using UK recommended depression case finding questions in antenatal care. Midwifery.

